# Characteristics of Yoga Practitioners, Motivators, and Yoga Techniques of Choice: A Cross-sectional Study

**DOI:** 10.3389/fpubh.2017.00184

**Published:** 2017-07-27

**Authors:** Shirley Telles, Sachin Kumar Sharma, Nilkamal Singh, Acharya Balkrishna

**Affiliations:** ^1^Patanjali Research Foundation, Haridwar, India

**Keywords:** yoga, characteristics of users, motivating factors, yoga techniques, survey, India

## Abstract

**Background:**

The characteristics of yoga practitioners and factors motivating people to practice yoga have been studied in the US and in Australia. This study aimed to determine the characteristics of yoga users in India, the factors that motivate them to practice yoga, and the yoga techniques of choice.

**Materials and methods:**

The study was a one-time, cross-sectional survey based on convenience sampling. Inclusion criteria were (a) a minimum of 1 week experience of yoga and (b) at least 10 years of age. 14,250 people received the survey. After excluding those who did not meet the inclusion criteria or filled in the survey incompletely or incorrectly, 5,157 respondents were included in the study.

**Results:**

Out of 5,157 respondents, there were more males (67.3%), aged between 21 and 44 years (33.7% of the sample surveyed), educated up to high school (62.5%), students (39.3%), and those who had between 1 and 12 months of experience in yoga (54.4%). The first most common reason to practice yoga for all respondents was physical fitness. Three of the remaining reasons to practice yoga differed significantly with age: (i) yoga for disease management (χ^2^ = 17.62, *p* < 0.005), (ii) yoga as a hobby (χ^2^ = 10.87, *p* < 0.05), and (iii) yoga based on the *guru’s* (teacher’s) instructions (χ^2^ = 20.05, *p* < 0.001). The yoga technique of choice [i.e., (i) *asanas* (χ^2^ = 23.17, *p* < 0.001), (ii) *pranayama* (χ^2^ = 19.87, *p* < 0.001), or (iii) meditation (χ^2^ = 9.64, *p* < 0.05)] differed significantly across age groups.

**Conclusion:**

In India, a yoga practitioner was more likely to be male, between 21 and 44 years of age, high school educated, and a student. The reasons to practice yoga and the yoga technique of choice differed significantly with age.

## Introduction

Yoga has multiple and diverse benefits (1). June 21 was declared the International Day of Yoga by the United Nations General Assembly, following a resolution from the Government of India (2). This declaration was based on the holistic approach to health and well-being through yoga. Hence, the declaration aimed to disseminate information about the overall benefits of yoga, for health (2).

The Associated Chambers of Commerce and Industries Social Development Foundation (India) conducted a survey (*n* = 1,100) which suggests that the intention of this resolution was met in India (3). There was a 30% increase in yoga practitioners in metropolitan cities following the first International Day of Yoga in India, between June 2015 and June 2016 (3). In the survey, the majority of yoga practitioners were students, young professionals, those in decision-making positions, and retired persons.

A survey was conducted in the US as a personal household interview, with 34,525 respondents (4). The survey was part of the 2012 National Health Interview Survey (4) and aimed at determining the reasons why people chose to meditate, the predictors for use, self-reported outcomes of meditation practice, meditation use within the life time and in a 12-month period, utilization patterns, and the prevalence of specific meditation practices. Another survey, which was part of the 2002 US National Health Interview Survey aimed at determining the characteristics of yoga users (5). There were 31,044 respondents, and the survey was administered as a face-to-face interview. The questions were intended to determine the prevalence of yoga users (found to be 5.1%, *n* = 1,593), characteristics of yoga users, medical reasons for practicing yoga, perceptions about the helpfulness, and the disclosure of use to medical professionals. A nation-wide survey was conducted in Australia as an online 30-min interactive web-based questionnaire, which was also intended to determine the characteristics of yoga practitioners, investigate the styles of yoga commonly practiced, the motivation to practice, perceived effects of practice, yoga-related injuries, as well as dietary and lifestyle choices (6). In both the US (4, 5) and Australia (6), yoga practitioners were more likely to be female, educated, and to be practicing yoga chiefly for physical well-being.

At least two surveys have been conducted in India. In a survey conducted in a city in the west of India (Mumbai), the aim was to determine whether university students were aware of scientific research on yoga (7). The survey was returned by 972 respondents, whose average age was 26 years and the ratio of males to females was 54.8%:45.2%. The other survey aimed to determine the barriers which yoga practitioners face when they are committed to practice yoga (8). The 280 participants had self-elected to join a 1 month residential course in yoga and participated in an online survey. Their average age was 34.5 years and the male to female ratio was 48.0:52.0.

To the best of our knowledge, there has been no survey in India to determine the characteristics of yoga users, their reasons for practicing yoga, and the yoga techniques they preferred. In June 2016 in India, several events preceded International Yoga Day to increase awareness about the multiple benefits of yoga.

The present survey on Indian respondents aimed at determining: (i) the characteristics of yoga users, (ii) the primary reason why people chose to practice yoga, and (iii) the yoga technique of their choice.

## Materials and Methods

### Sample

**Figure 1:**
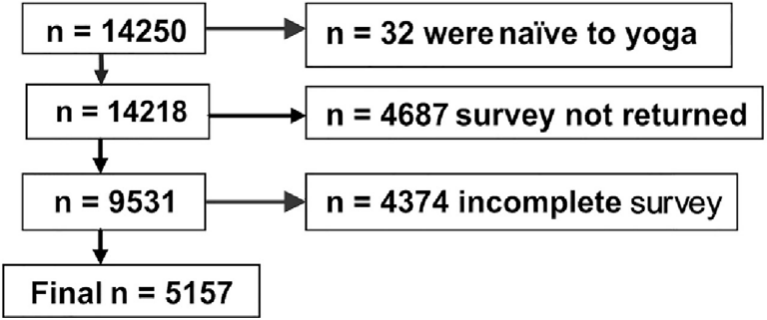
Details about the number of respondents.

The respondents to the survey had come from all over India to participate in or observe a public event on yoga. To be included in the survey participants had to have (i) a minimum of 1 week experience of yoga practice and (ii) completed at least 10 years of age. 14,250 individuals received the survey. Excluding those who did not meet the inclusion criteria or did not complete the survey correctly, 5,157 respondents formed the sample (Figure 1). The baseline characteristics of these respondents are provided in Table 1. Their ages ranged between 10 and 91 years. The gender ratio (M:F in %) was 67.3:32.7. They had varying levels of education, and experience in yoga, as well as different occupations. The signed informed consent of each respondent was taken as part of the survey.

**Table 1:** Baseline characteristics of the participants.

Characteristics	Details
**Age (years)**	
Group mean age (SD)	33.4 (17.3)
Age range	10–91
**Gender: male:female (actual values), % values**	3,469:1,688, 67.3:32.7
**Years of education (%)**	
10 years and less	39.2
11–12 years	23.2
13–15 years	24.2
16 years and above	13.2
**Experience of yoga (%)**	
Less than a month	7.1
1–12 months	54.4
13–60 months	23.8
60 months and above	14.7

### Design

The study was a one-time cross-sectional survey. The total number of participants exceeded 25,000. The selected sample size was 15,000. This was not based on statistical calculations but on pragmatic considerations. Administering the survey simultaneously to 15,000 participants posed practical difficulties. Hence, it was decided to administer the survey when participants were seated in smaller groups. Buses were arranged to transport participants from a specific point to the venue. There were 50 participants in each bus. Hence, 300 buses were selected so as to get the required number of 15,000 respondents. No standard methods of randomization were used to select buses, which is a definite limitation of the survey. The survey was administered by a volunteer (one volunteer/stationary bus). The volunteers had been briefed about the survey questions. There were no specific considerations for participants to enter a bus and entry was free. The study had prior clearance from the ethical committee of Patanjali Research Foundation (approval no. PRF/16/0023), which was formed based on the guidelines of the Indian Council of Medical Research (9).

### The Event and Yoga Protocol

The event was in New Delhi on June 19, 2016 in a public space with multiple entrances. The event was organized by a yoga institution in north India (*viz*., Patanjali Yogpeeth). Entry was free and unrestricted. The grounds were easily accessible. Participants were given yoga mats to practice yoga or to observe others practice. Naïve-to-yoga participants were instructed to be observers. The event involved yoga practices from the common yoga protocol developed by the Ministry of AYUSH, Government of India (10), devised to be practiced across India on International Yoga Day. These techniques were considered relatively safe for all yoga practitioners. Contraindications for practice were mentioned. The 45-min protocol included a universal prayer, loosening exercises, yoga postures, yoga breathing techniques, and meditation. The details are given in Table 2.

**Table 2:** The 45-min yoga protocol devised by the Ministry of AYUSH, Government of India (10).

Sl. no.	Type of practice	Duration (min)
1	Prayer in any meditative posture with *Namaskara Mudra* and ending with Yoga *Mudrasana*	2
2	*Sadilaja/Chaalan Kriyas*/loosening practices (neck, shoulders, trunk, and knees movements)	6
3	Light exercises and specific yoga postures (*asanas*) Standing postures *Taadaasana* (palm tree pose) *Vrikshaasana* (tree pose) *Pada-hastaasana/Uttaanaasana* (hand to foot pose/standing forward bend pose) *Ardha chakraasana* (standing backward bend pose) *Trikonaasana* (triangle pose) Sitting postures *Bhadraasana/Baddhakonaasan* (butterfly pose) *Vajrasana/Veerasana* (thunderbolt pose/hero pose) *Ushtraasana (Ardha* for beginners*)*(camel pose) *Shashankaasan* (rabbit pose) *Utthana Mandukasana* (stretched frog pose) *Marichyaasana/Vakraasana* (ray of light pose/curved pose) Prone lying postures *Makaraasana* (crocodile pose) *Bhujangaasana* (cobra pose) *Shalabhaasana* (locust pose) Supine lying postures *Setubandhasana* (bridge pose) *Utthanapaadaasana* (leg raised pose) *ArdhaHalasana* (half plough pose) *PawanaMuktaasana* (wind relieving pose) Shavaasan (corpse pose)	18
4	*Kapalabhati* (3 cycles of 40 strokes each) (kapala = forehead; bhati = shining; rapid, forceful exhalations) Each cycle will be followed deep breathing	3
5	*Pranayama* *NadiShodhana/AnulomaViloma Pranayama* (alternate nostril breathing) (5 rounds) *Sheetali Pranayama* (cooling breath) (5 rounds) *Bhramari Pranayama* (bee breathing) (5 rounds)	6
6	*Dhyana*/Meditation in any Meditative Posture (eyes closed) and hands in *Jnana/Gyana Mudra*	8
7	End of the yoga Practice Session with a *Sankalp*	2
	Total duration	45

### The Survey

The survey was written in both English and Hindi. It had three parts: (i) Details about the respondents: age, gender, education, occupation, and experience in yoga, (ii) the primary reason why the participants wanted to practice yoga, and (iii) the yoga practice they liked the most. Participants were asked to sign an informed consent form. The second part of the survey, i.e., the primary reason to practice yoga had 10 possible responses with examples. These were (i) arranged in alphabetical order in English, (ii) given serial numbers, and (iii) the sequence of the 10 options was randomized using a standard randomizer (www.randomizer.org). Each person was asked to select one option which they rated as their primary reason to practice yoga. The authors’ experience with yoga practice and research (from 1991 till the present time) (11–13) was taken into account to develop the survey.

For the third part of the survey, the yoga practices were selected based on (i) the eight limbs (*asthang* in *Sanskrit*) of yoga, described by the sage Patanjali (*Circa* 900 B.C.) (14) and (ii) the yoga practices commonly taught in India (15). There were six options written in English and given their *Sanskrit* name. These 6 options were then randomized as described above for the 10 options in the second part of the survey. The final sequence is presented in Table 3. Each respondent was asked to select their first choice out of the six options.

**Table 3:** Questions asked in the survey.

Part of the survey	Section	Options
Part II	Motivators to practice yoga based on the question: why do you want to practice yoga?	Disease management (e.g., diabetes, hypertension) Belief with no specific reasons (that is as a part of faith) Mental development (e.g., increase memory) As a hobby Physical strength (e.g., muscle strength) Spiritual growth (e.g., *sadhana*) Because my friends practice it Emotional health (e.g., relief from depression) Because my *guru* says I should Belief based on scientific knowledge (e.g., books or research publications)
Part III	Choice of yoga practice based on the question: which practice of yoga do you like the most?	*Yamas* and *Niyamas* *Asanas* *Pranayamas* *Kriyas* *Mudras* and *Bandhas* Meditation

*The sequence for options for both Part II and Part III has been randomized. Part I included socio-demographic data sheet*.

*Participants were instructed to select one option that was most appropriate for part II and similarly for Part III*.

### Data Extraction

Survey sheets were sorted for different characteristics such as age, gender, years of education, occupation, and experience of yoga. Under each category, the number of respondents who were practicing yoga for a specific reason was noted. Similarly, the yoga practice of choice was noted for respondents under each category, for example, male and female. Handling this volume of data is prone to error. Hence, each entry was checked by two researchers, independently.

### Data Analyses

The values were converted as a percentage of the whole within each category. The percentages have been used in the discussion. They have been provided for the reasons to practice yoga (Table 4) and for the yoga practices of choice (Table 5). Also, the percentage values were used to carry out separate Chi square tests to detect differences between-groups in the responses to the two questions (i.e., the primary reason to practice yoga and the yoga practice of choice), under the categories: age, gender, years of education, occupation, and experience in yoga.

**Table 4:** The 10 motivators to practice yoga for the 5,157 respondents.

			Reasons to practice yoga									
				2	3	4	5	6	7	8	9	10
			
Characteristics		Total sample	Yoga for disease management*	Blind faith in yoga	Yoga for mental development	Yoga as a hobby*	Yoga for physical fitness	Yoga for spiritual growth	Because friends practice yoga	Yoga for emotional health	Because my *guru* (teacher) says*	Belief based on scientific knowledge
1. Gender	Male	3,469	438 (12.6%)	148 (4.3%)	347 (10.0%)	191 (5.5%)	1,667 (48.1%)	295 (8.5%)	59 (1.7%)	27 (0.8%)	116 (3.3%)	181 (5.2%)
	Female	1,688	227 (13.4%)	63 (3.7%)	179 (10.6%)	100 (5.9%)	895 (53.0%)	99 (5.9%)	14 (0.9%)	16 (0.9%)	40 (2.4%)	55 (3.3%)
2. Age (in years)	10–12	208	13 (6.3%)	9 (4.3%)	28 (13.5%)	30 (14.4%)	88 (42.3%)	4 (1.9%)	1 (0.5%)	2 (1.0%)	26 (12.5%)	7 (3.3%)
	13–20	1,642	129 (7.9%)	66 (4.0%)	205 (12.5%)	115 (7.0%)	833 (50.7%)	90 (5.5%)	38 (2.3%)	17 (1.0%)	69 (4.2%)	80 (4.9%)
	21–44	1,739	199 (11.4%)	66 (3.8%)	187 (10.8%)	75 (4.3%)	930 (53.5%)	141 (8.1%)	17 (1.0%)	17 (1.0%)	32 (1.8%)	75 (4.3%)
	45–59	1,081	203 (18.7%)	42 (3.9%)	76 (7.0%)	50 (4.6%)	502 (46.4%)	112 (10.4%)	13 (1.2%)	06 (0.7%)	23 (2.1%)	54 (5.0%)
	60 and above	487	121 (24.8%)	28 (5.7%)	30 (6.2%)	21 (4.3%)	209 (42.9%)	47 (9.7%)	04 (0.8%)	01 (0.3%)	06 (1.2%)	20 (4.1%)
3. Education (in years)	10 years and below	2,023	273 (13.5%)	85 (4.2%)	207 (10.2%)	133 (6.6%)	986 (48.7%)	132 (6.5%)	39 (1.9%)	14 (0.8%)	83 (4.1%)	71 (3.5%)
	11–12 years	1,202	147 (12.2%)	47 (3.9%)	145 (12.1%)	74 (6.2%)	588 (48.9%)	82 (6.8%)	16 (1.3%)	15 (1.3%)	41 (3.4%)	47 (3.9%)
	13–15 years	1,250	153 (12.2%)	53 (4.2%)	117 (9.4%)	55 (4.4%)	645 (51.6%)	114 (9.1%)	11 (0.9%)	9 (0.7%)	26 (2.1%)	67 (5.4%)
	16 years and above	682	92 (13.5%)	26 (3.8%)	57 (8.4%)	29 (4.3%)	343 (50.3%)	66 (9.7%)	7 (1.0%)	5 (0.6%)	6 (0.9%)	51 (7.5%)
4. Experience in yoga	Less than 1 month	365	47 (12.9%)	18 (4.9%)	35 (9.6%)	25 (6.8%)	181 (49.6%)	19 (5.2%)	10 (2.8%)	5 (1.4%)	15 (4.1%)	10 (2.7%)
	1–12 months	2,805	320 (11.4%)	110 (3.9%)	335 (11.9%)	158 (5.6%)	1,465 (52.2%)	162 (5.8%)	46 (1.7%)	21 (0.8%)	90 (3.2%)	98 (3.5%)
	13–60 months	1,228	171 (13.9%)	53 (4.3%)	114 (9.3%)	69 (5.6%)	593 (48.3%)	114 (9.3%)	12 (1.0%)	12 (1.0%)	33 (2.7%)	57 (4.6%)
	60 months and above	759	127 (16.7%)	30 (4.0%)	42 (5.5%)	39 (5.1%)	323 (42.6%)	99 (13.0%)	5 (0.7%)	5 (0.7%)	18 (2.3%)	71 (9.4%)
5. Occupations	Students	2,028	159 (7.8%)	77 (3.8%)	267 (13.2%)	159 (7.8%)	1,007 (49.7%)	106 (5.2%)	39 (1.9%)	20 (1.0%)	95 (4.7%)	99 (4.9%)
	Employed	1,070	143 (13.4%)	47 (4.4%)	97 (9.1%)	47 (4.4%)	534 (49.9%)	103 (9.6%)	15 (1.4%)	8 (0.8%)	22 (2.0%)	54 (5.0%)
	Self-employed	844	141 (16.7%)	33 (3.9%)	65 (7.7%)	38 (4.5%)	373 (44.2%)	107 (12.7%)	11 (1.3%)	6 (0.7%)	23 (2.7%)	47 (5.6%)
	Homemakers	781	132 (16.9%)	29 (3.7%)	59 (7.5%)	29 (3.7%)	452 (57.9%)	44 (5.6%)	5 (0.7%)	5 (0.7%)	8 (1.0%)	18 (2.3%)
	Retired	182	38 (20.9%)	15 (8.2%)	13 (7.1%)	9 (4.9%)	83 (45.6%)	10 (5.5%)	2 (1.1%)	1 (0.6%)	2 (1.1%)	9 (5.0%)
	Not mentioned	252	52 (20.6%)	10 (3.9%)	25 (9.9%)	9 (3.6%)	113 (44.8%)	24 (9.5%)	1 (0.5%)	3 (1.2%)	6 (2.4%)	9 (3.6%)

*χ^2^ p < 0.05 across age groups.

**Table 5:** The 6 yoga practices provided as options in a random sequence to the 5,157 respondents.

			Yoga practices					
			1	2	3	4	5	6
		
Characteristics		Sample size	*Yama-niyams*	Asanas*	Pranayama^*,@^	*Kriyas*	*Mudras-Bandhas*	Meditation*
1. Gender	Male	3,469	194 (5.6%)	842 (24.3%)	1493 (43.0%)	79 (2.3%)	44 (1.2%)	817 (23.6%)
	Female	1,688	69 (4.1%)	501 (29.7%)	733 (43.4%)	33 (2.0%)	13 (0.7%)	339 (20.1%)

2. Age (in years)	10–12	208	10 (4.8%)	102 (49.0%)	58 (27.9%)	3 (1.4%)	2 (1.0%)	33 (15.9%)
	13–20	1,642	66 (4.0%)	545 (33.2%)	463 (28.2%)	43 (2.6%)	18 (1.1%)	507 (30.9%)
	21–44	1,739	83 (4.7%)	407 (23.4%)	803 (46.2%)	46 (2.7%)	18 (1.0%)	382 (22.0%)
	45–59	1,081	72 (6.7%)	194 (17.9%)	628 (58.1%)	12 (1.1%)	14 (1.3%)	161 (14.9%)
	60 and above	487	32 (6.6%)	95 (19.5%)	274 (56.3%)	8 (1.6%)	05 (1.0%)	73 (15.0%)

3. Education (in years)	10 years	2,023	110 (5.4%)	592 (29.3%)	773 (38.2%)	51 (2.5%)	30 (1.5%)	467 (23.1%)
	11–12 years	1,202	72 (6.0%)	293 (24.4%)	476 (39.6%)	29 (2.4%)	11 (0.9%)	321 (26.7%)
	13–15 years	1,250	59 (4.7%)	286 (22.9%)	621 (49.7%)	22 (1.8%)	9 (0.7%)	253 (20.2%)
	16 years and above	682	22 (3.2%)	172 (25.2%)	356 (52.2)	10 (1.5%)	7 (1.0%)	115 (16.9%)

4. Experience in yoga	Less than 1 month	365	22 (6.0%)	96 (26.3%)	123 (33.7%)	8 (2.2%)	2 (0.6%)	114 (31.2%)
	1–12 months	2,805	127 (4.5%)	805 (28.7%)	1,101 (39.3%)	69 (2.5%)	34 (1.1%)	669 (23.9%)
	13–60 months	1,228	61 (5.0%)	305 (24.8%)	583 (47.5%)	28 (2.3%)	16 (1.3%)	235 (19.1%)
	60 months and above	759	53 (7.0%)	137 (18.0%)	419 (55.2%)	7 (0.9%)	5 (0.7%)	138 (18.2%)

5. Occupations	Students	2,028	79 (3.9%)	688 (33.9%)	584 (28.8%)	51 (2.5%)	23 (1.2%)	603 (29.7%)
	Employed	1,070	65 (6.1%)	224 (20.9%)	547 (51.1%)	25 (2.4%)	15 (1.4%)	194 (18.1%)
	Self-employed	844	59 (7.0%)	169 (20.0%)	439 (52.0%)	15 (1.8%)	10 (1.2%)	152 (18.0%)
	Homemakers	781	39 (4.9%)	175 (22.4%)	419 (53.6%)	17 (2.3%)	5 (0.7%)	126 (16.1%)
	Retired	182	9 (4.9%)	45 (24.7%)	98 (53.8%)	0 (0.0%)	1 (0.7%)	29 (15.9%)
	Not mentioned	252	12 (4.8%)	42 (16.7%)	139 (55.2%)	4 (1.6%)	3 (1.1%)	52 (20.6%)

*χ^2^ p < 0.05 for asanas, pranayama, and meditation across age groups.

*^@^χ^2^ p < 0.05 pranayama across occupation*.

## Results

The first most common reason for all respondents to practice yoga was physical fitness. Three motivators, i.e., (i) disease management (χ^2^ = 17.62, *p* < 0.005), (ii) yoga as a hobby (χ^2^ = 10.87, *p* < 0.05), and (iii) practicing yoga because my *guru* says to practice it (χ^2^ = 20.05, *p* < 0.001) differed significantly between the five age ranges (i.e., 10–12, 13–20, 21–44, 45–59, and 60 years and above).

The yoga practice of choice also differed significantly between the different age groups: (i) *asanas (*χ*^2^* = 23.17, *p* < 0.001), (ii) *pranayamas* (χ*^2^* = 19.87, *p* < 0.001), and meditation (χ^2^ = 9.64, *p* < 0.05). *Pranayamas* as the practice of choice differed significantly with occupations (χ*^2^* = 9.62, *p* < 0.05).

## Discussion

5,157 respondents completed a survey in June 2016 in New Delhi, India. There were more males (67.3%) compared to females (32.7%).

The maximum respondents were adults between 21 and 44 years (33.7%), followed by adolescents between 13 and 20 years (31.7%) (16). Children between 10 and 12 years were least (4.0%) and seniors 60 years and above were 9.4%.

Most yoga practitioners had between 8 and 10 years of education (39.2%). Ten years is equal to a basic school leaving certificate. The smallest percentage had at least 16 years of education (13.2%). This corresponds to post-graduation and further studies.

The respondents could be broadly categorized into five occupations. Students formed the highest percentage (39.3%) while retired persons were the fewest (3.5%). There is an increased interest in young people to be physically fit and the number of young people choosing to practice yoga has increased all over India (3). A contributing factor could partly be that younger persons would be expected to be more enthusiastic about such an outdoor event and also would find the venue more accessible.

Among the respondents, the maximum number had experience of yoga ranging from 1 to 12 months (54.4%). This could be related to the fact that the maximum respondents were students.

Hence, in the present survey in India, more yoga practitioners were male, aged between 21 and 44 years with education between 8 and 10 years, students, and with 1 and 12 months experience of yoga. These results contrasted with those obtained in the US, where yoga practitioners were more often female, between 40 and 64 years of age (4) or with an average age of 39.5 years (5), with a minimum of college education, and a greater likelihood of being White, non-Hispanic, and Caucasian (4, 5). In Australia too, participants were more often female, with an average age of 41 years and were tertiary educated (6). The gender ratio in this study resembles that of a survey carried out in west India in Mumbai, on 972 practitioners (7) where males exceeded females (54.8:45.2). The results differed from the results of another survey conducted in India on 280 persons who had self-selected to join for a 1-month yoga residential course (8). Among the 280 respondents, the number of males and females was comparable, i.e., 48:52. In this study, despite the large number of participants, there was adequate space and security measures; hence, it is unlikely that these factors influenced the participation of females. The other factors (e.g., 8–10 years of education, more students practicing yoga in India) which differed between surveys conducted in India versus the US or Australia may be attributed to aims and aspirations of young people in a developing economy such as India (17).

We mentioned 10 reasons in the survey that could motivate participants to practice yoga. The first most common reason for all respondents to practice yoga was physical fitness. Selecting physical fitness as a primary reason to practice yoga is comparable to the results of the survey in Australia (6) and surveys in the US (4, 5).

Three motivators (i.e., practicing yoga as a hobby, practicing based on a *guru’s* instructions and for disease management) differed significantly between the five age ranges. The 10- to 12-year age group chose “practicing yoga as a hobby” which was significantly higher in comparison to the percentages of the four other age groups (i.e., 13–20, 21–44, 45–59, and 60 years and above). For children to practice yoga as a hobby is a healthy trend which should be encouraged (18). The reasons why children chose “yoga as a hobby” as a motivating factor could be many; there is an increased attempt to introduce yoga in schools in India to alleviate stress and reduce the growing incidence of childhood obesity (19). It is important to mention here that despite yoga having been introduced in certain schools in India, yoga is not treated as a subject for which students have a regular examination. This could allow children to learn yoga without a feeling of performance anxiety and stress. Apart from this in India, there are several sports persons and other celebrities who promote yoga, which may have influenced the way in which children perceive yoga practice (20). Practicing yoga “based on a *gurus’s* instructions” was also significantly higher in children between 10 and 12 years age group compared to the percentages of the four other age groups (i.e., 13–20, 21–44, 45–59, and 60 years and above). This was an interesting trend in pre-teens and could be related to observations that Asian parents tended to be more authoritarian than their Caucasian counterparts (21). This also suggests that despite an increased awareness of freedom which pre-teens have in many cultures (22), in India traditional values appear to prevail in a large percentage of pre-teens. It is debatable whether it is ideal that children of this age should be so dependent on instructions from their *guru*. Some parents allow children of this age too much of the wrong kind of freedom or they offer freedom before the adolescent is ready to accept it. Other parents cling too tightly, denying pre-teens both the responsibilities they require to develop maturity and the opportunities they need to make choices and accept their consequences. The present results suggest that the attitude of these pre-teens requires to be reviewed. Respondents aged between 21 and 60 years and above selected disease management as their second most common reason to practice yoga. This could be related to the increased incidence of non-communicable diseases such as diabetes and hypertension in middle-aged persons in India (23, 24), as well as a growing awareness that yoga and healthy lifestyle choices can help in the management of these diseases (25).

The third part of the survey provided information about the yoga practice the participants were most likely to choose. The factors which significantly influenced the choice of yoga practice were age and occupation.

Children between 10 and 12 years and adolescents (13–20 years) selected *asanas* as their first choice of practice. A possible reason is that most children prefer being active to sitting still as is required for *pranayama* and meditation practice (26). Also, *asanas* are known to improve physical fitness (27). This is supported by the findings of the survey, i.e., children and adolescents chose yoga to improve physical fitness as their primary reason to practice yoga.

Adults chose *pranayama* as their first choice and, *asanas* as their second choice of practice. The practice of *asanas* requires flexibility (28), while *pranayama* can also be practiced by those who are less mobile (29) or less flexible. It is well known that both mobility and flexibility reduce with age (30). This may explain why adults selected *pranayama* as their first choice of practice whereas for children and adolescents *asanas* were their first choice of practice.

The results of this survey show a significant difference between the percentages of respondents who selected *pranayama* as their primary choice of practice for different occupations. Out of five occupations, respondents from four categories of occupations selected *pranayama* as their first choice of practice. These were (i) employed, (ii) self-employed, (iii) retired, and (iv) homemakers. *Pranayama* practice is more popular in India than elsewhere, and specific sets of practices (15) and techniques such as *Sudarshan Kriya* Yoga (31) are practiced widely across the country. Apart from this, middle aged and older adults who have not been physically active often find *pranayama* easier to practice (32). Students’ first two most common choices were *asanas* and meditation. Hence, the finding that *pranayama* as a primary choice of practice varies with the type of occupation, could be related to other factors, such as the age of the respondents and their level of physical activity and fitness. None of the respondents selected *yamas*/*niyamas, mudras*/*bandhas*, or *kriyas* as their first choice of yoga practice.

In summary, more yoga practitioners were male, aged between 21 and 44 years, with education between 8 and 10 years, students, and with 1 and 12 months experience of yoga. The first most common reason to practice yoga for all respondents was physical fitness. Three of the remaining reasons to practice yoga differed significantly with age: (i) yoga for disease management, (ii) yoga as a hobby, and (iii) yoga based on the *guru’s* (teacher’s) instructions. The yoga technique of choice [i.e., (i) *asanas*, (ii) *pranayama*, or (iii) meditation] differed significantly across age groups.

All respondents selected physical fitness as their primary reason to practice yoga. Three motivators (i.e., practicing yoga as a hobby, disease management, and practicing based on a *guru’s* instructions) differed significantly between the five age ranges. The choice of yoga practice was influenced by age and occupation significantly.

The findings of this survey are limited by the method of sampling. Despite this limitation, the results represent people from across the Indian subcontinent. A further systematically conducted survey would help to substantiate the findings.

## Ethics Statement

The study had prior clearance from the ethical committee of Patanjali Research Foundation (approval no. PRF/16/0023) which was formed based on the guidelines of the Indian Council of Medical Research.

## Author Contributions

SS collected and analyzed the data. ST drafted the manuscript. SS and NS assisted in compiling the revised manuscript, ST, AB, and SS contributed to the study design and interpretation of results. ST and SS will act as guarantors.

## Conflict of Interest Statement

The authors declare that the research was conducted in the absence of any commercial or financial relationships that could be construed as a potential conflict of interest.
